# Trends of neural tube defects in urban China and effects of socio-demographic factors, 2013–2022: a descriptive analysis

**DOI:** 10.1136/bmjph-2024-001489

**Published:** 2025-07-16

**Authors:** Xiao Cheng, Chao Meng, Gabriel L Galea, Lirong Nie, Duoduo Wang, Shuangbo Xia, Yuan Wei, Jufen Liu, Zhiwen Li

**Affiliations:** 1 Institute of Reproductive and Child Health/National Health Commission Key Laboratory of Reproductive Health, Peking University, Beijing, China; 2 Department of Epidemiology and Biostatistics, School of Public Health, Peking University, Beijing, China; 3 Department of Maternal Health Care, Beijing Haidian Maternal and Child Health Hospital, Beijing, China; 4 Developmental Biology and Cancer Department，UCL Great Ormond Street Institute of Child Health, University College London, London, UK; 5 Department of Gynaecology and Obstetrics, Peking University Third Hospital, Beijing, China

**Keywords:** Epidemiology, Public Health, Epidemiologic Factors, trends

## Abstract

**Background:**

Periconceptional folic acid supplementation has progressively reduced the prevalence of neural tube defects (NTDs) in rural Northern China, which had a high baseline. Long-term epidemiological trends have not been comprehensively assessed in urban China with a lower baseline.

**Methods:**

Data from a hospital-based birth-defect surveillance system in Haidian District between 2013 and 2022 were used. The prevalence of NTDs by different characteristics was analysed. χ^2^, Pearson’s tests and Joinpoint regression analyses were used to compare the prevalence of NTDs by year and explore trends.

**Results:**

363 732 births and 258 NTDs were recorded in Beijing’s Haidian District from 2013 to 2022. NTD prevalence did not change significantly: it decreased in the first 4 years, then increased and subsequently hovered around 8.14/10 000. Stalling of NTD prevention is superimposed on changing maternal socio-demographics, including increasing education and self-reported regular use of folic acid in NTD-affected pregnancies. Maternal age of NTD-affected pregnancies also increased markedly from 2013 to 2022. To test which factors might alter NTD risk, we compared NTD-affected pregnancies with those affected by non-NTD congenital malformations. Compared with non-NTD malformations, advanced maternal age and multiple birth are independent risk factors for NTDs (adjusted OR: 1.776 and 2.513, respectively). Multiparity and regular folic acid use are protective factors (adjusted OR: 0.040 and 0.556).

**Conclusions:**

The accessibility of maternal folic acid in urban China needs to be evaluated, and it is necessary to investigate biological differences which may render some NTDs folic acid resistant. Meanwhile, the implementation of mandatory fortification of foods with folic acid is imperative.

**WHAT IS ALREADY KNOWN ON THIS TOPIC**

- Some neural tube defects (NTDs) can be prevented by maternal folic acid intake and some socio-demographic factors may confound preventative effects of folic acid, but a systematic analysis of NTD epidemiology in urban China is lacking.

**WHAT THIS STUDY ADDS**

- Despite an increase in regular folic acid intake in urban China, changes in the socio-demographic profile of women, such as increasing maternal age, may offset the impact of folic acid intake, resulting in minimal change in NTD prevalence over time.

**HOW THIS STUDY MIGHT AFFECT RESEARCH, PRACTICE OR POLICY**

- Both socio-demographic and biological factors may hinder NTD prevention. This study supports the need to evaluate the accessibility of maternal folic acid use in urban China beyond retrospectively self-reported indicators and mechanistically investigate biological differences which may render some NTDs folic acid resistant.

## Introduction

 Neural tube defects (NTDs), including anencephaly, spina bifida and encephalocele, are severe congenital malformations of the central nervous system which originate during embryonic development when the neural tube develops abnormally. NTDs remain a major cause of perinatal mortality and represent a significant clinical and public health challenge, affecting around 10/10 000 births globally.^1^ Prevalence estimates differ dramatically, both within and between countries. For instance, in the early 2000s NTD prevalence in parts of rural Northern China was 100/10 000, which was 10 times higher than that in urban China.^2 3^ Intense research over the past decade has begun to explain the differences in prevalence, including epidemiological risk factors such as indoor pollution and biological associations due to genetic polymorphisms.^4 5^ The extremely high prevalence of NTDs in rural Northern China motivated public health interventions to prevent these conditions through supplementation with folic acid—the only agent clearly demonstrated to reduce NTDs risk across populations.

Folic acid’s prevention of NTDs was proven in multinational trials in the 1980s. The MRC Vitamins trial studied high-risk individuals who already had an NTD-affected pregnancy and were therefore all multiparous, with a median age of 27 years. Of those randomised to receive folic acid before pregnancy, 0.6% had a recurrent NTD-affected pregnancy compared with 3.6% of those who did not receive it.^6^ Although this trial clearly showed that a subset of NTD recurrences cannot be prevented by folic acid supplementation even under clinical trial conditions, it also provided evidence of a dramatic reduction in risk from a high baseline. That core conclusion of the trial, followed by trials demonstrating prevention of NTD occurrence rather than recurrence,^7 8^ motivated many countries around the world to implement folic acid fortification of foodstuffs, or to provide more targeted supplementation for women of childbearing age coupled with antenatal education campaigns.^9^,^11^ Both approaches have proven effective to different extents in different countries. For example, NTD prevalence dropped from over 100/10 000 births prior to supplementation to almost 20/10 000 births a decade later in rural China.^12 13^

What is lacking is a systematic long-term analysis of epidemiological trends in NTD prevalence in more urban areas of China, which are likely to be more comparable to developed countries in Europe and North America. The UK, which until recently lacked folic acid provision, observed fluctuations in NTD prevalence without marked temporal trends over the past 20 years.^14^ Canada, which implements folic acid fortification, observed a significant increase in NTD prevalence due to changes in maternal risk factors.^15^ Well-established risk factors for NTDs include maternal antiepileptic medication use and pre-gestational diabetes.^16 17^ Other putative maternal risk factors are less clearly understood. For example, advanced maternal age (≥35 years old) has been paradoxically associated with increased NTD risk in some populations and lower risk in others.^15 18 19^ The association between maternal age and risk of non-chromosomal congenital malformations is unlikely to be a simple linear one,^20^ likely due to various social and biological risk factors.

The rarity of NTDs, prenatal diagnosis challenges and elective termination of pregnancy complicate risk factor studies. Several countries and geographical regions have ongoing congenital malformation surveillance strategies, sometimes limited by non-standardised methodologies or definitions, and changes in implementation over time.^21^ The national programme of hospital-based congenital malformation surveillance in China circumvents many of these issues, providing unique epidemiological data. Specifically, the Beijing Maternal and Child Health Network Information System is a long-running surveillance system which includes a detailed questionnaire completed by trained healthcare professionals documenting information on both fetal and maternal factors. These data are rigorously checked at both district and city level by dedicated quality control staff, requiring contributing institutes to clarify records whenever needed.^22^ The healthcare system in China also ensures very high levels of population engagement with the service due to the requirement for early registration with a hospital to access perinatal services including a birth certificate.

Congenital malformation surveillance data in China show sustained NTD prevalence reduction in rural areas from a high baseline after the national folic acid supplementation campaign.^12^ A similar reduction was expected in urban areas, such as the Haidian District of Beijing, motivating the initiation of an equivalent folic acid supplementation campaign 1 year later than in rural areas of Northern China. However, we observe that NTD prevalence in Haidian has not decreased over the past decade despite a marked increase in the proportion of mothers with NTD-affected pregnancies reporting regular folic acid intake. We explore demographic changes which may confound increased use of folic acid and discuss their potential impact on NTD prevention, along with potential biological explanations.

## Methods

### Congenital malformations surveillance system

Data from a hospital-based birth-defect surveillance system in the district were analysed in this study. The Beijing Birth Defects Registration Card (for Obstetrics and Gynecology) tracks congenital malformations prevalence, including NTDs for national maternal and child health monitoring. Unlike the national version, which mainly uses hard copy paper data input and reporting, congenital malformation registration information in Beijing was merged into the Beijing Maternal and Child Health Network Information System in 2022, which is a more comprehensive resource with advanced online access, in-built record checks, convenient management visibility and quality control.

Congenital malformations reports include two parts, mostly from obstetric reports (13 gestational weeks to 7 days post partum) and paediatric reports (8 days post partum to 1 year old). Here we only include data from obstetric reports. All live births or stillbirths of 28 or more complete gestational weeks and pregnancy terminations at any gestational age following the prenatal diagnosis of NTDs were included from 18 community health service centres, midwifery agencies and children’s hospitals in Haidian District. In healthcare institutions, which are the first-level and direct data collector, specially assigned and trained staff fill out uniform forms and congenital malformations registration, as well as reporting individual information for each case. Important diagnostic reports and clinical images are uploaded for better assessment of congenital malformations. The reporting time for individual cases is based on the date of delivery of the fetus, not the date of death or reduction, even in twin pregnancies where one has already died or undergone reduction.

Data quality is strictly controlled. Once case information is inputted into the system, reviewers from the institution, district and municipal levels confirm the diagnosis. Besides the regular inputting and review, there are self-inspections for any omission using the hospital medical record system to check based on the International Statistical Classification of Diseases and Related Health Problems 10th revision (ICD10) code in discharge diagnosis. Then the district and county conduct 1–4 inspections per year for missing or under-reporting of congenital malformations at institutions which use the hospital’s medical record input system. The Beijing Maternal and Child Health Network Information System also conducts under-reporting investigations on perinatal deaths. A previous study revealed that the under-reporting rate of NTDs decreased from 12.4% in 2010 to 6.6% in 2012.^23^ In Beijing and other urban regions of China, integrated prenatal diagnosis management further reduced under-reporting.^24^ Starting from 2022, Beijing requires that prenatal screening and prenatal diagnosis cases be recorded in the maternal and child information system and can also be used for under-reporting investigation based on the above cases. Finally, there are on-site spot checks at the municipal level at least once a year correspondingly. Beijing Haidian Maternal and Child Health Hospital and Peking University Third Hospital were the designated district-level and municipal-level professional institutions.

### Diagnosis of NTDs and quality control

Three ICD10 code subtypes—anencephaly, Q00; spina bifida, Q05; and encephalocele, Q01—were included. Detailed diagnostic information is recorded in the system for prenatal diagnosis. Ultrasonic examination and blood biochemical tests are conducted in the first trimester. In the current study, all congenital malformations are categorised as NTDs, NTD-related and NTD-independent congenital malformations.^25^

NTD-related defects include hydrocephalus, omphalocele, gastroschisis, congenital deformities of the hip, limb reduction defects, cleft lip or/and cleft palate and congenital defects of the urinary system (ICD10 codes Q03, Q79.2, Q79.3, Q65, Q71-72, Q35-37 and Q64, respectively). NTD-independent defects are syndactyly, polydactyly, diaphragmatic hernia, anorectal atresia or stenosis and oesophageal atresia or stenosis (ICD10 codes Q69, Q70, Q79.0, Q42 and Q39, respectively). Congenital malformations and other diseases were systematically double-checked in the hospital medical record system.

As the data set we used is the congenital malformation surveillance system which did not record births without congenital malformations due to the separate register system for women with healthy live births and with birth defects in China.^22^ To test whether maternal age is a risk factor for NTDs, we then compared NTD-affected pregnancies with those affected by non-NTDs, which we called NTD-independent congenital malformations instead. We consider these NTD-independent congenital malformations a ‘control’ group, assuming averaging of population characteristics when multiple unrelated congenital malformations are combined.

### Folic acid supplementation and demographic information

Folic acid supplementation was recorded from a maternal questionnaire. ‘Regular’ intake was defined as taking 400 µg folic acid daily from 3 months before pregnancy until the first trimester of pregnancy (third months after pregnancy) for at least 80% of total days. Those who self-reported taking folic acid less than 80% of total days or less than taking 400 µg folic acid daily were recorded as not taking folic acid regularly, and those who self-reported not taking folic acid were recorded as not. Other demographic information, including maternal age, education, residence, parity, gestational weeks, number of fetuses, fetal sex and birth outcomes, was collected through the system.

### Statistical analysis

Pre-perinatal (NTD cases diagnosed before 28 gestational weeks) and perinatal (NTDs diagnosed at 28 or more gestational weeks) prevalence were calculated. χ^2^ test, Pearson’s correlation test and Fisher’s exact test were performed using SPSS (V.26.0, SPSS Inc, Chicago, Illinois, USA) and R (V.4.4.0) to compare the prevalence of NTDs by subtype, year and gestational weeks. The Joinpoint Regression Program (V.5.0.2. May 2023; Statistical Research and Applications Branch, National Cancer Institute) was used to analyse the trends of prevalence of NTDs, average maternal ages, parity, maternal education and folic acid use. After using univariate analysis to select different characteristics, a multivariate regression model was used to compare the selected risk factors related to NTDs or NTD-independent congenital malformations. Two-tailed p≤0.05 was considered statistically significant.

## Results

### NTD prevalence did not decrease consistently between 2013 and 2022 in urban Beijing

**Figure 1:**
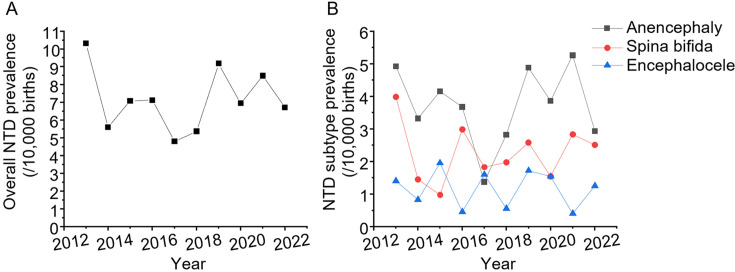
Prevalence of neural tube defects (NTDs) by subtypes, Haidian District, Beijing, 2013–2022: (A) overall NTD prevalence and (B) NTD subtype prevalence.

A total of 258 cases of NTDs were recorded among 363 732 births in Haidian between 2013 and 2022 (online supplemental table 1). NTD prevalence did not decrease consistently over this decade (figure 1A). Specifically, the prevalence of NTDs initially decreased from 10.32/10 000 in 2013 to 4.80/10 000 in 2017, but then increased to 9.18/10 000 in 2019 and hovered around 8.14/10 000 over the next 3 years (figure 1A, online supplemental table 2). Fluctuations in prevalence were observed in all three NTD subtypes studied. The prevalence of anencephaly decreased from 4.93/10 000 in 2013 to 1.37/10 000 in 2017, then fluctuated between 2018 and 2022, whereas the prevalence of spina bifida decreased from 3.99/10 000 in 2013 to 1.83/10 000 in 2017, then increased to 2.51/10 000 after 2018 (figure 1B). The prevalence of encephalocele hovered around 1.18/10 000 throughout the period (figure 1B). Overall, the prevalence of anencephaly was the highest among the three subtypes, accounting for nearly half of all NTDs, while the prevalence of encephalocele was the lowest (figure 1B).

The majority of NTDs were diagnosed early, as indicated by a high proportion (89.8% overall) terminated before 28 gestational weeks (online supplemental table 1). A significant trend was observed in the proportion of fetuses delivered at ≥28 weeks gestation, decreasing over time from 27.3% in 2013 to 6.2% in 2022 (online supplemental figure 1A, online supplemental table 2). This trend may be due to an anomalously large number of spina bifida cases in 2013, with six individuals diagnosed and all born alive. Overall, the proportion of NTD cases terminated, born alive (predominantly spina bifida, online supplemental table 1) or which resulted in neonatal death, did not change significantly over the past decade (online supplemental table 2).

### A large and increasing proportion of NTD-affected pregnancies are in women who report regular folic acid intake

**Figure 2:**
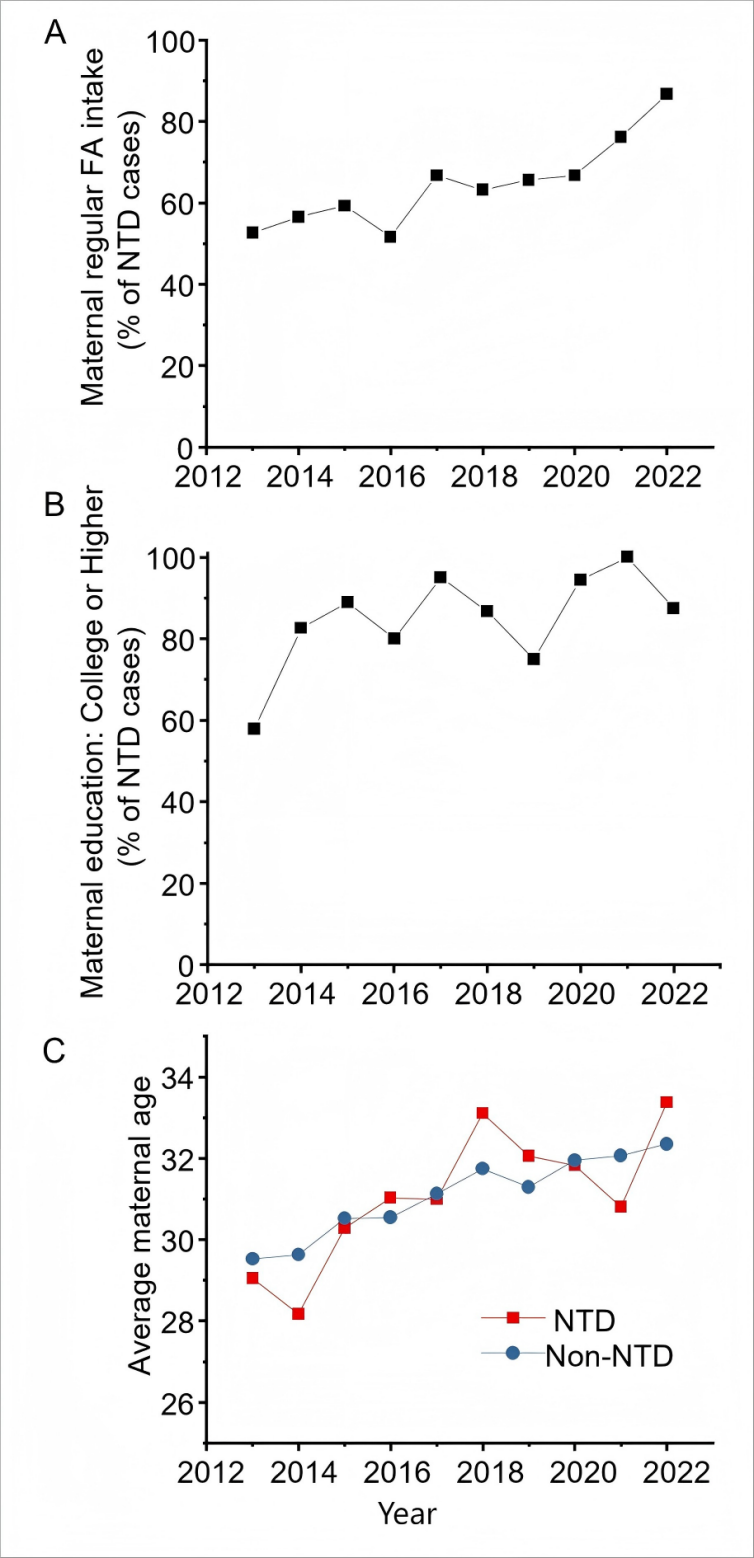
Maternal characteristics by birth defects and year, Haidian District, Beijing, 2013–2022. (**A**) Proportion of mothers taking folic acid (FA) regularly, 2013–2022. (**B**) Proportion of mothers with higher maternal education in neural tube defect (NTD)-affected pregnancy, 2013–2022. (**C**) Average maternal age among women with birth defects, 2013–2022.

We hypothesised that the lack of temporal changes in NTD prevalence could be due to consistent folic acid use over time. Contrary to this hypothesis, the proportion of mothers with NTD-affected pregnancies who reported regular folic acid intake increased significantly over the past 10 years, from 52.6% in 2013 to 86.7% in 2022 (figure 2A, online supplemental table 2). Folic acid use did not differ significantly between maternal age groups, parity or number of fetuses (table 1). However, individuals with NTD-affected pregnancies who had college education or above were more likely to report regular use of folic acid: 66% of mothers with NTD-affected pregnancies who report higher education levels (college or above) took folic acid regularly compared with 45.2% of those with lower education levels (table 1). Compared with the cases who take folic acid irregularly, NTDs are less prevalent in those who take folic acid regularly (online supplemental figure 2). The education level of mothers with NTD-affected pregnancies increased significantly over the time period analysed: 57.9% reported college or higher education in 2013 compared with 87.5% in 2022 (figure 2B, online supplemental table 1). This suggests that despite documentable protective effects of regular folic acid use, increased maternal folic acid use—associated with higher education—has not translated into a reduction in NTD prevalence, potentially due to changes in other risk factors.

**Table 1:** Characteristics of neural tube defects by condition of taking folic acid

Characteristics	Taking folic acid supplementation			P value
	Regularly	Not regularly	No	
Age group				0.137
<30	58 (55.8)	26 (25.0)	20 (19.2)	
30–34	63 (71.6)	18 (20.5)	7 (7.9)	
≥35	32 (60.4)	14 (26.4)	7 (13.2)	
Education				0.032*
High school or lower	19 (45.2)	13 (30.9)	10 (23.9)	
College or above	126 (66.0)	42 (22.0)	23 (12.0)	
Parity				0.252
Nulliparous	96 (66.7)	31 (21.5)	17 (11.8)	
Multiparous	57 (56.4)	27 (26.7)	17 (16.9)	
Number of fetuses				0.320
Singleton	136 (61.0)	55 (24.7)	32 (14.3)	
Multiple birth	17 (77.3)	3 (13.6)	2 (9.1)	

* p<0.05.

### Maternal age of NTD-affected pregnancies increases markedly in Haidian

The congenital malformations surveillance system used does not record information on maternal risk factors such as pre-gestational diabetes, but does record maternal age. The average maternal age of those with NTD-affected pregnancies increased markedly over the past decade (figure 2C). The proportion of mothers ≥35 years old increased significantly from 4.6% in 2013 to 37.5% in 2022 (online supplemental table 2). NTD subtypes were not significantly different between maternal age groups (online supplemental table 1). Given the study period spans a change in national policy—from a one-child to a two-child and then three-child policy—we tested whether parity changed over time, finding no significant trend (online supplemental table 2). It is therefore unlikely that cessation of the one-child policy has resulted in a significant increase in maternal age due to higher parity.

### Advanced maternal age and first parity increase the risk of NTDs compared with other congenital malformations irrespective of the protective effect of regular folic acid intake

In the Haidian population, a very small proportion of NTD-affected pregnancies were reported in mothers <25 years old (6% compared with 30.6% in a recent UK study^26^). However, comparisons between NTD-affected pregnancies cannot assess whether maternal age is a risk factor for NTDs. The surveillance system used does not record births without congenital malformations which could be compared as ideal ‘control’. However, it does record other congenital malformations which are not NTDs. In previous work, we identified subtypes of congenital malformations which are significantly over-represented in fetuses with NTDs,^25^ potentially reflecting shared genetic or environmental risk factors, and types of congenital malformations not associated with NTDs.

We therefore compared NTD-affected pregnancies with those affected by defects which are not epidemiologically associated with NTDs (table 2). Consistent with this, we observed a smaller proportion of NTD-affected pregnancies in mothers who reported regular folic acid intake compared with the corresponding proportions for other congenital malformations (table 2, online supplemental table 3), supporting a protective role of regular folic acid use selectively for NTDs. Twinning is a known risk factor for NTDs^27^and we observe a higher proportion of multiple pregnancies than singleton pregnancies among those affected by NTDs compared with other congenital malformations (online supplemental table 3).

**Table 2:** Maternal characteristics of NTDs compared with NTD-independent birth defects, Haidian District, Beijing, 2013–2022, n (%)

Characteristics	NTD cases(n=258)	NTD-independent BD cases(n=2656)	P value
Taking FA			<0.001*
No	34 (13.9)	288 (11.4)	
Not regular	58 (23.7)	374 (14.8)	
Regularly	153 (62.4)	1865 (73.8)	
Maternal age (mean±SD)	30.86±4.29	30.95±4.35	0.800
Maternal age			0.263
<30	104 (42.5)	990 (38.9)	
30–34	88 (35.9)	1050 (41.3)	
≥35	53 (21.6)	503 (19.8)	
Education			0.471
High school or lower	42 (17.9)	493 (19.9)	
College or above	192 (82.1)	1983 (80.1)	
Parity			<0.001*
Nulliparous	152 (58.9)	169 (6.4)	
Multiparous	106 (41.1)	2487 (93.6)	
Number of fetuses			0.007*
Singleton	224 (91.1)	2409 (95.1)	
Multiple birth	22 (8.9)	124 (4.9)	

Comparisons between NTD cases and other BD cases using the t-test (maternal age) and Pearson’s χ2 test (all categorical characteristics).

* p<0.05.

BD, birth defect; FA, folic acid; NTD, neural tube defect.

Pregnancies with non-NTD congenital malformations were analysed to test maternal age trends and relative risk. Average maternal age increased between 2013 and 2022 for NTD-independent congenital malformations as it has for those with NTDs (figure 2C), suggesting a wider trend towards greater maternal age across the population. However, NTD cases were over-represented in mothers ≥35 years old compared with corresponding proportions for pregnancies affected by other congenital malformations (online supplemental table 3), suggesting advanced maternal age increases NTD risk in this population. Paradoxically, the opposite association was observed with maternal parity: multiparous accounted for 41.1% of NTD-affected pregnancies compared with 93.6% of NTD-independent congenital malformations (table 2). This suggests NTDs are comparatively more likely than other congenital malformations to occur in the first pregnancy.

Univariate comparisons between NTDs and non-NTD congenital malformation cohorts were extended using multivariable logistic regression. This identified advanced maternal age (≥35 years old) and multiple birth as independent risk factors for NTDs, whereas multiparity and regular folic acid use are protective factors compared with NTD-independent congenital malformations (online supplemental table 2).

## Discussion

Prevention strategies for NTDs have not significantly changed over the past decade. Much of the evidence for the effectiveness of folic acid in preventing these conditions is based on interventions carried out in previously non-supplemented populations and with exposure to risk factors not necessarily representative of modern urban centres.^2 3^ In China, education on folic acid use is communicated through public health campaigns, such as the Program of Supplementation of Folic Acid for the Prevention of NTDs, which was launched as the major public health project by the Ministry of Health in 2009,^28^ and it was included in the National Basic Public Health Service Project in China in 2019 and then incorporated into the National Basic Public Service Standards by the Chinese government on 30 July 2023.^29^ Northern China is dominated by the Han ethnic group, which accounts for 95% of our data set. Hence, it provided equal opportunities for women of childbearing age across the country regardless of ethnicity. The district (urban) or county (rural) health administrative department organises the same distribution and management of folic acid through various channels such as premarital healthcare, pre-pregnancy healthcare and prenatal healthcare. The residential district (urban) or town (rural) health officer is responsible for the distribution of folic acid, guiding their standardised use and scientific management, and follow-up process of folic acid for women preparing to conceive.^28 30 31^ The effectiveness of these campaigns in Haidian is shown by numerous women reporting regular folic acid intake despite NTD-affected pregnancies. The preventative effect of regular folic acid use is also evident when comparing NTD-affected pregnancies with those affected by other congenital malformations.

Monitoring the prevalence of NTDs periodically in populations is needed to guide the implementation of effective fortification policies for the prevention of NTDs.^30^ From the continuous monitoring in Northern China, we found that the compliance of supplementation for folic acid in Beijing is higher than in other regions in Northern China, Shanxi Province, where the prevalence is higher than that in Beijing.^3 32^ In 2013–2016, 51.6% of mothers with NTDs reported folic acid intake in Shanxi,^32^ and 84% of mothers with NTDs reported folic acid intake in Beijing during the same period. Hence, regular accessibility and high compliance of folic acid supplementation are key for NTD prevention, which indeed requires continuous monitoring, although other mechanisms that affect folate metabolism and absorption are still worth investigating.^33 34^

The monitoring in Europe shows that the prevalence of NTDs in some European countries is higher^35^ than that reported in Beijing, which is possibly related to the differences in racial, folic acid supplementation and genetic background,^34^ although there were still other unknown factors which need to be explored further.^14 36^ In countries such as the USA and Canada, which have implemented the fortification of folic acid programme for nearly two decades, the prevalence of NTD is very low, which is almost 22% lower than urban China.^37^ Meanwhile, European countries such as France and the UK have not yet implemented mandatory folic acid programmes by 2023, and the prevalence fluctuates before entering a bottleneck period; however, it was higher than the same period in the USA.^14 38^ Notably, other non-NTD birth defects such as congenital heart malformation are also increasing in prevalence, which is similar to Europe and the USA.^35 37^ This may be due to the improvements of prenatal and postnatal diagnosis,^39^ maternal diseases and antipsychotic medication exposure.^20 40^ At the same time, a previous study in China also showed that the prevalence of some non-NTD anomalies increases with higher maternal age.^30^ Given the increasing evidence that folic acid may cause unwanted epigenetic changes,^41^ these will also be important avenues for future research and require mechanistic studies. Nonetheless, stable NTD prevalence, despite increasing regular maternal use of folic acid in Haidian, is concerning. Both socio-demographic and biological factors may hinder NTD reduction.

Through the comparison of the ratio of NTD with non-NTD malformations by folic acid intake status, we can find that a smaller proportion of mothers with NTD-affected pregnancies reported taking folic acid regularly than those with non-NTD birth defects. This protective effect reinforces the importance of folic acid supplementation combined with educational campaigns to promote effective use, particularly in the absence of staple foodstuff fortification. Higher maternal education correlates with folic acid intake. Increasing maternal education is one measure of changing population demographics, which may both decrease and increase NTD risk. The link between higher maternal education and folic acid use has previously been observed in other populations.^42^ Other populations have also seen socio-demographic changes associated with increasing maternal obesity and substance misuse, which may be related to low education and increased NTD risk.^17 43^ Healthcare access and technologies have also improved over time, although this is unlikely to be a confounding factor in the highly developed Haidian District of Beijing. As in the rest of China, policies regulating reproductive behaviour have also changed in Haidian during the study period. Introduction of the two-child policy from 2016 and three-child policy from 2020, motivated by decreasing birth rates, may have resulted in individual parents choosing to have additional children, but has not resulted in a substantial population-level shift towards multiparity. Changes in reproductive behaviour may also be related to maternal education: previous studies have associated increased time spent in education with greater maternal age at first pregnancy.^42^

Some changes in maternal demographics may alter biological risk factors. Advanced maternal age is a well-established risk factor for chromosomal abnormalities,^43^ some of which may be associated with NTDs.^2 3^ However, the interaction between maternal age and risk of congenital malformations is complex and unlikely to represent a simple linear association. For example, a recent study in Hungary showed both young (<20 years old) and advanced (≥35 years old) maternal age is associated with increased risk of non-chromosomal anomalies.^20^ Ymaternal ages are only less than 4% in the Haidian population due to the national legal age for marriage of 20 years old for women.^31^ Maternal age is associated with a rapid decline in the production of healthy and high-quality oocytes, resulting in reduced fertility in women older than 35 years of age.^44^ There is also an increase in life and work pressure as maternal age increased, which may be related to unfavourable birth outcomes, including NTDs. This increase in life and work stress may manifest^45^ as prenatal anxiety,^46^ smoking, drinking or antipsychotic medication use.^40^

It is widely known that folic acid can significantly reduce the risk of NTD, and advanced maternal age may also be related to NTDs. Under the combined effects of the two factors mentioned above, the prevalence of NTDs in urban Beijing did not decrease consistently. However, NTDs are a multifactorial disorder, which are partly caused by genetic variation (e.g. in *MTHFR*),^34^ DNA methylation, histone modification (in particular acetylation) or chromatin remodelling,^33 34^ and environmental factors.^36 47^ A complex combination of genetic and environmental interactions^48^ could also affect NTDs. However, those mechanisms mentioned above still require further research. With the global ageing population and the fertility change nowadays, social behaviour and demographic characteristics such as advanced maternal age, increased education and urbanisation will become increasingly prominent, which deserves further attention. What is less clear is whether NTDs which occur in women who regularly use folic acid starting before pregnancy are biologically different from those which can be prevented by regular folic acid use since around 30% of the NTDs that occur are folate-resistant.^36 47^ For example, to our knowledge, the effectiveness of folic acid at preventing NTDs has not been compared between mothers in different age groups.

This study was subject to limitations. Since healthy infants without congenital malformations (considered to be the ideal 'control' group) are not included in the surveillance system, we selected birth defects not epidemiologically associated with NTDs and combined them to form a control group, which was assumed to have population-average characteristics. This approach may induce bias. Further studies are required to investigate the genetic or environmental risk factors between these defects and healthy live births.

In summary, this study revealed that increasing maternal folic acid use and education have not persistently reduced NTD prevalence in Haidian between 2013 and 2022, which is similar to the trend of NTD changes in other countries that have not implemented fortification of folic acid programmes, such as the UK.^49^ Socio-demographic factors of mothers with pregnancies affected by NTDs, or with other malformations, have changed over this period, particularly as evidenced by the marked increase in maternal age. NTDs and non-NTD congenital malformations have distinct risk factors consistent with different biological mechanisms and potential for prevention by folic acid. These data support the need to evaluate the accessibility of maternal folic acid use in urban China beyond retrospectively self-reported indicators and mechanistically investigate biological differences which may render some NTDs folic acid resistant. The fortification of folic acid is a method worth considering, but its accessibility in China is still worth further research due to cost, technical method and other reasons. This does not argue against the fact that the existing folic acid supplementation programme has significantly reduced the prevalence of NTDs in urban China.

## Supplementary material

10.1136/bmjph-2024-001489 online supplemental file 1

10.1136/bmjph-2024-001489 online supplemental figure 1

10.1136/bmjph-2024-001489 online supplemental figure 2

## Data Availability

Data are available upon reasonable request.
