# Supplementary material for: Trends of neural tube defects in urban China and effects of socio-demographic factors, 2013–2022: a descriptive analysis
Source: BMJ Public Health. 2025 Jul 16;3(2):e001489. doi: 10.1136/bmjph-2024-001489 (PMC12273093; doi:10.1136/bmjph-2024-001489)
Supplement: online supplemental file 1 [file bmjph-3-2-s001.docx]

**Trend of Neural Tube Defects in Urban China and Effect of Social-demographic Factors, 2013-2022: A descriptive analysis**

**Supplemental Table legend**

Supplemental Table 1. Characteristics with NTDs by subtypes in Haidian District, Beijing, China,2013-2022

Supplemental Table 2 Characteristics with NTDs by years in Haidian District, Beijing, China, 2013–2022

Supplemental Table 3 Logistic regression analysis of maternal demographic factors of NTDs compared with other birth defects, Haidian District, Beijing, 2013-2022

**Supplemental Table 1. Characteristics with NTDs by subtypes in Haidian District, Beijing, China, 2013–2022, n (%)**

| Variables | Total NTDs  (*n*=258) | Anencephaly  (*n*=133) | Spina bifida  (*n*=82) | Encephalocele  (*n*=43) | *P* value |
| --- | --- | --- | --- | --- | --- |
| Taking folic acid^#^ |  |  |  |  | 0.864 |
| Regularly | 153 (62.5) | 77 (61.6) | 49 (62.8) | 27 (64.3) |  |
| Not Regularly | 58 (23.7) | 33 (26.4) | 16 (20.5) | 9 (21.4) |  |
| None | 34 (13.8) | 15 (12.0) | 13 (16.7) | 6 (14.3) |  |
| Maternal Age |  |  |  |  | 0.982 |
| <30 | 117 (45.4) | 61 (45.9) | 38 (46.3) | 18 (41.9) |  |
| 30–34 | 88 (34.1) | 46 (34.6) | 27 (32.9) | 15 (34.9) |  |
| ≥35 | 53 (20.5) | 26 (19.5) | 17 (20.8) | 10 (23.2) |  |
| Education^#^ |  |  |  |  | 0.703 |
| High school or lower | 42 (18.0) | 20 (16.7) | 13 (17.6) | 9 (22.5) |  |
| College or above | 192 (82.0) | 100 (83.3) | 61 (82.4) | 31 (77.5) |  |
| Parity |  |  |  |  | 0.104 |
| Nulliparous | 152 (58.9) | 86 (64.7) | 41 (50.0) | 25 (58.1) |  |
| Multiparous | 106 (41.1) | 47 (35.3) | 41 (50.0) | 18 (41.9) |  |
| Gestational weeks at delivery or termination |  |  |  |  | <0.001* |
| <28 | 227 (88.0) | 127 (95.5) | 60 (73.2) | 40 (93.0) |  |
| ≥28 | 31 (12.0) | 6 (4.5) | 22 (26.8) | 3 (7.0) |  |
| Number of fetus |  |  |  |  | 0.159 |
| Singleton | 224 (91.1) | 111 (88.8) | 76 (96.2) | 37 (88.1) |  |
| Multiple birth | 22(8.9) | 14 (11.2) | 3 (3.8) | 5 (11.9) |  |
| Sex |  |  |  |  | <0.001^*^ |
| Male | 100 (38.8) | 47 (35.3) | 36 (43.9) | 17 (39.5) |  |
| Female | 71 (27.5) | 19 (14.3) | 40 (48.8) | 12 (27.9) |  |
| Unknown | 87 (33.7) | 67 (50.4) | 6 (7.3) | 14 (32.6) |  |
| Pregnancy outcome^#^ |  |  |  |  | <0.001^*^ |
| Live | 19 (7.8) | 1 (0.8) | 15 (18.9) | 3 (7.2) |  |
| Termination | 219 (89.8) | 118 (95.9) | 62 (78.6) | 39 (92.8) |  |
| Neonate death | 6 (2.4) | 4 (3.3) | 2 (2.5) | 0 (0) |  |

*NTDs* neural tube defects

^*^ *P* ＜0.05 ^#^ excluding missing value

**Supplemental Table 2**. **Characteristics with NTDs by years in Haidian District, Beijing, China,2013-2022, *n* (%)**

| Variables | Total |  | 2013 | 2014 | 2015 | 2016 | 2017 | 2018 | 2019 | 2020 | 2021 | 2022 | *P* value |
| --- | --- | --- | --- | --- | --- | --- | --- | --- | --- | --- | --- | --- | --- |
| Prevalence (1/10,000 births) | 7.09 |  | 10.32 | 5.60 | 7.09 | 7.12 | 4.80 | 5.36 | 9.18 | 6.95 | 8.50 | 6.70 | 0.086 |
| Taking Folic Acid^#^ |  |  |  |  |  |  |  |  |  |  |  |  | 0.021* |
| Regularly | 153 (62.5) |  | 20 (52.6) | 13 (56.5) | 16 (59.3) | 16 (51.6) | 14 (66.7) | 12 (63.2) | 21 (65.6) | 12 (66.7) | 16 (76.2) | 13 (86.7) |  |
| Not regularly | 58 (23.7) |  | 12 (31.6) | 7 (30.4) | 8 (29.6) | 8 (25.8) | 4 (19.1) | 5 (26.3) | 6 (18.8) | 5 (27.8) | 1 (4.8) | 2 (13.3) |  |
| None | 34 (13.8) |  | 6 (15.8) | 3 (13.1) | 3 (11.1) | 7 (22.6) | 3 (14.2) | 2 (10.5) | 5 (15.6) | 1 (5.5) | 4 (19.0) | 0 (0) |  |
| Maternal Age |  |  |  |  |  |  |  |  |  |  |  |  | ＜0.001^*^ |
| ＜30 | 117 (45.4) |  | 27 (61.3) | 20 (74.1) | 15 (51.7) | 14 (45.2) | 8 (38.1) | 4 (21.1) | 11 (34.4) | 8 (44.4) | 8 (38.1) | 2 (12.5) |  |
| 30–34 | 88 (34.1) |  | 15 (34.1) | 4 (14.8) | 11 (37.9) | 9 (29.0) | 8 (38.1) | 7 (38.7) | 12 (37.5) | 3 (16.7) | 11 (52.4) | 8 (50.0) |  |
| ≥35 | 53 (20.5) |  | 2 (4.6) | 3 (11.1) | 3 (10.3) | 8 (25.8) | 5 (23.8) | 8 (42.2) | 9 (28.1) | 7 (38.9) | 2 (9.5) | 6 (37.5) |  |
| Education ^#^ |  |  |  |  |  |  |  |  |  |  |  |  | 0.001^*^ |
| High school or lower | 42 (18.0) |  | 16 (42.1) | 4 (17.4) | 3 (11.1) | 6 (20.0) | 1 (5.0) | 2 (13.3) | 7 (25.0) | 1 (5.6) | 0 (0) | 2 (12.5) |  |
| College or above | 192 (82.0) |  | 22 (57.9) | 19 (82.6) | 24 (88.9) | 24 (80.0) | 19 (95.0) | 13 (86.7) | 21 (75.0) | 17 (94.4) | 19 (100) | 14 (87.5) |  |
| Parity |  |  |  |  |  |  |  |  |  |  |  |  | 0.708 |
| Nulliparous | 152 (58.9) |  | 22 (50.0) | 21 (77.8) | 19 (65.5) | 18 (58.1) | 11 (52.4) | 7 (36.8) | 16 (50.0) | 11 (61.1) | 18 (85.7) | 9 (56.3) |  |
| Multiparous | 106 (41.1) |  | 22 (50.0) | 6 (22.2) | 10 (34.5) | 13 (41.9) | 10 (47.6) | 12 (63.2) | 16 (50.0) | 7 (38.9) | 3 (14.3) | 7 (43.7) |  |
| Gestational weeks at delivery or termination |  |  |  |  |  |  |  |  |  |  |  |  | 0.030^*^ |
| ＜28 | 227 (88.0) |  | 32 (72.7) | 25 (92.6) | 28 (96.6) | 27 (87.1) | 20 (95.2) | 15 (78.9) | 27 (84.4) | 17 (94.4) | 21 (100) | 15 (93.8) |  |
| ≥28 | 31 (12.0) |  | 12 (27.3) | 2 (7.4) | 1 (3.4) | 4 (12.9) | 1 (4.8) | 4 (21.1) | 5 (15.6) | 1 (5.6) | 0 (0) | 1 (6.2) |  |
| Number of fetus |  |  |  |  |  |  |  |  |  |  |  |  | 0.563 |
| Singleton | 224 (91.1) |  | 34 (89.5) | 22 (95.7) | 23 (85.2) | 30 (96.8) | 17 (80.9) | 17 (89.5) | 29 (90.6) | 18 (100) | 19 (90.5) | 15 (93.8) |  |
| Multiple birth | 22 (8.9) |  | 4 (10.5) | 1 (4.3) | 4 (14.8) | 1 (3.2) | 4 (19.1) | 1 (10.5) | 3 (9.4) | 0 (0) | 2 (9.5) | 1 (6.2) |  |
| Pregnancy outcome ^#^ |  |  |  |  |  |  |  |  |  |  |  |  | 0.080 |
| Live | 19 (7.8) |  | 9 (23.7) | 0 (0) | 1 (3.8) | 3 (9.7) | 1 (4.8) | 1 (5.3) | 2 (6.5) | 1 (5.6) | 0 (0) | 1 (6.3) |  |
| Termination | 219 (89.8) |  | 28 (73.7) | 22 (96.6) | 25 (96.2) | 27 (87.1) | 20 (95.2) | 17 (89.4) | 27 (87.0) | 17 (94.4) | 21 (100) | 15 (93.7) |  |
| Neonate death | 6 (2.4) |  | 1 (2.6) | 1 (3.4) | 0 (0) | 1 (3.2) | 0 (0) | 1 (5.3) | 2 (6.5) | 0 (0) | 0 (0) | 0 (0) |  |

*NTDs* neural tube defects

^*^ *P* ＜0.05 # excluding missing value

**Supplemental Table 3. Logistic regression analysis of maternal demographic factors of NTDs compared with NTD-independent birth defects, Haidian District, Beijing, 2013-2022**

| Variables ^†^, | aOR | 95%CI | *P* value |
| --- | --- | --- | --- |
| Taking folic acid |  |  |  |
| No | 1 |  |  |
| Not regular | 1.304 | 0.736-2.107 | 0.324 |
| Regularly | 0.556 | 0.319-0.801 | 0.013* |
| Maternal age |  |  |  |
| <30 | 1 |  |  |
| 30-34 | 1.01 | 0.713-1.432 | 0.955 |
| ≥35 | 1.776 | 1.179-2.674 | 0.006* |
| Parity |  |  |  |
| Nulliparous | 1 |  |  |
| Multiparous | 0.04 | 0.029-0.055 | <0.001* |
| Number of fetus |  |  |  |
| Singleton | 1 |  |  |
| Multiple birth | 2.513 | 1.440-4.385 | 0.001* |

*aOR* adjusted odds ratio

† Variable(s) entered: Parity, Taking folic acid, maternal age, Pregnancy types, maternal age

* *P*<0.05
