# Supplementary figures and images for: Trends of neural tube defects in urban China and effects of socio-demographic factors, 2013–2022: a descriptive analysis
Source: BMJ Public Health. 2025 Jul 16;3(2):e001489. doi: 10.1136/bmjph-2024-001489 (PMC12273093; doi:10.1136/bmjph-2024-001489)

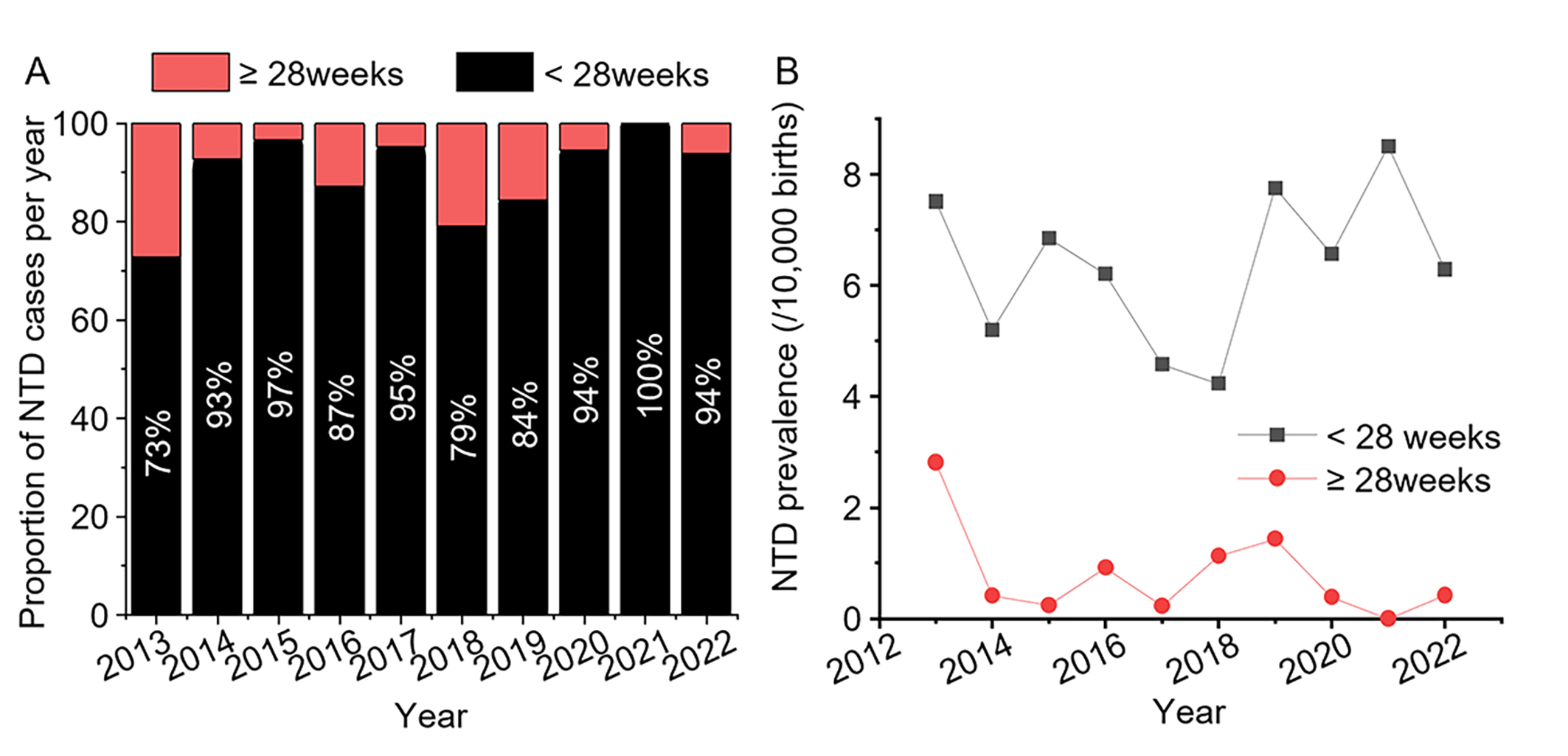

Supplement: online supplemental figure 1 [file bmjph-3-2-s002.jpg]

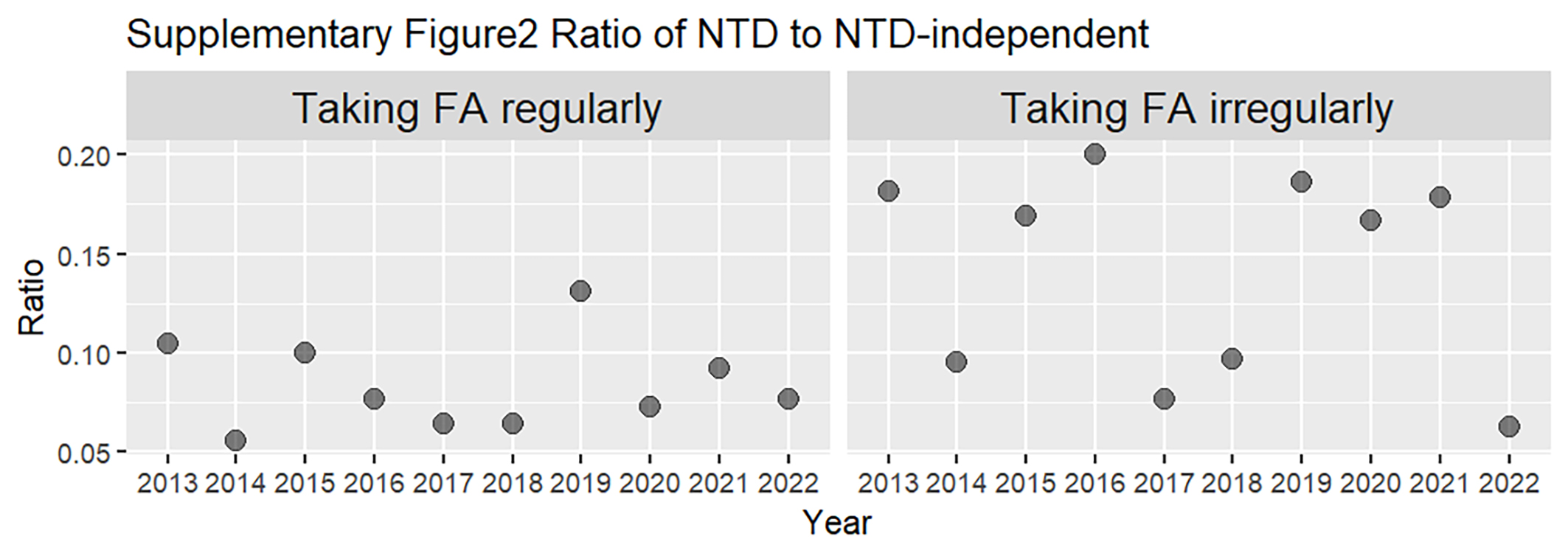

Supplement: online supplemental figure 2 [file bmjph-3-2-s003.jpg]
